# Relationship between Glioblastoma Dose Volume Parameters Measured by Dual Time Point Fluoroethylthyrosine-PET and Clinical Outcomes

**DOI:** 10.3389/fneur.2017.00756

**Published:** 2018-01-22

**Authors:** Maciej Harat, Bogdan Małkowski, Izabela Wiatrowska, Roman Makarewicz, Krzysztof Roszkowski

**Affiliations:** ^1^Department of Radiotherapy, The Franciszek Lukaszczyk Oncology Centre, Bydgoszcz, Poland; ^2^Department of Positron Emission Tomography and Molecular Imaging, Nicolaus Copernicus University in Toruń, Ludwik Rydygier Collegium Medicum, Bydgoszcz, Poland; ^3^Department of Nuclear Medicine, The Franciszek Lukaszczyk Oncology Centre, Bydgoszcz, Poland; ^4^Department of Medical Physics, The Franciszek Lukaszczyk Oncology Center, Bydgoszcz, Poland; ^5^Department of Oncology and Brachytherapy, Nicolaus Copernicus University in Toruń, Ludwik Rydygier Collegium Medicum, Bydgoszcz, Poland; ^6^Department of Oncology and Brachytherapy, The Franciszek Lukaszczyk Oncology Centre, Bydgoszcz, Poland; ^7^Department of Oncology, Radiotherapy and Ginecologic Oncology, Faculty of Health Sciences, Nicolaus Copernicus University Toruń, Bydgoszcz, Poland

**Keywords:** glioblastoma multiforme, fluoroethylthyrosine-PET, radiotherapy, progression-free survival, dose map

## Abstract

Glioblastoma multiforme (GBM) is highly invasive. Despite irradiation with wide margins, GBM usually recurs in-field. Recent *in vitro* data have suggested that progression might be promoted by sublethal irradiation. Fluoroethylthyrosine-PET (FET-PET) can be used to detect glioblastoma invasion not apparent on MRI. We therefore performed a retrospective analysis of a prospective clinical study to examine whether glioblastoma outcomes depend on dose volume parameters measured by MRI and FET-PET. Twenty-three patients were prospectively recruited to a study examining the role of dual time point FET-PET in the treatment planning of GBM radiotherapy. The dose delivered to the site of recurrence was subdivided into suboptimal-dose (SOD) and high-dose (HD) areas. Types of progression were defined for correlation with dosimetric parameters including V100% of gross tumor volume (GTV)_PET_, GTV_PETMRI_, and GTV_MRI_. The HD area did not cover the entire GTV_PETMRI_ in any case. Recurrences were significantly more frequent in the SubD area (chi-squared test, *p* = 0.004). There was no relationship between increasing dose volume and progression. The V100% for GTV_PET_ and progression-free survival (PFS) was positively correlated (Spearman’s rho 0.417; *p* = 0.038). Progression is more common in areas with suboptimal dosing. Dose heterogeneity within GTV_PET_ may be responsible for shorter PFS.

## Introduction

Glioblastoma multiforme (GBM) is the most common glial tumor in adults. The standard treatment for GBM is surgery followed by radiotherapy with concomitant adjuvant chemotherapy (1). The highly invasive and extensive infiltrative growth pattern of GBM limits curative resections (2).

Recurrence after treatment most frequently occurs within 20 mm of the border of the primary focus seen in postoperative MRIs. Irradiating the tumor area and surrounding edema or the entire brain does not improve outcomes (3, 4), and dose escalation in areas originally identified on the MRI does not improve local control, resulting in approximately 80% central (in-field) failure (5, 6).

Precise target volume definition is crucial in conformal or intensity-modulated radiotherapy, because underdosing the tumor may result in treatment failure. On the other hand, whole-brain dose escalation may lead to unacceptable toxicity. Difficulties in defining areas at risk of recurrence might be in part responsible for failed escalation studies in GBM patients.

Preclinical studies have shown that sublethal irradiation of glioma cells results in the formation of a greater number of tumor satellites in rat brains *in vivo* associated with enhanced matrix metalloproteinase-2 and reduced tissue inhibitor of metalloproteinases-2 expression (7). Similarly, sublethal irradiation of glioma cell lines *in vitro* induces glioma invasion in a brain tumor model (8). It is also hypothesized that irradiated normal brain tissues might promote tumor invasion and angiogenesis, promoting glioma recurrence.

18F-fluoroethylthyrosine-PET (FET-PET) can be used to improve the accuracy of determining the extent of GBM infiltration. Regardless of the MRI sequence used, stereotactic biopsies confirm the presence of tumor cells in 40% of areas that appear unchanged on MRI (2). Combining PET with a cell proliferation marker shows that tumor infiltration extends 35 mm outside the area shown in both MRI sequences (9).

T1-gad sequences in MRI and FET-PET studies achieve a sensitivity and specificity of 93 and 94% for determining glioblastoma extent, respectively (10). Dynamic PET with FET is recommended for grading gliomas, prognostication of untreated gliomas, and differentiation of metastasis and recurrence from radiation-induced changes (11). We recently showed that FET-PET at dual time points (dtpFET-PET) precisely defines the most probable sites of failure (12). Moreover, biological tumor volumes defined by FET-PET, especially in dual time point acquisition compared with T1-weighted sequences, are significantly larger. As a consequence, they may accumulate a more inhomogeneous and sublethal dose.

In clinical practice, dose inhomogeneity of between 95 and 107% within the planning treatment volume (PTV) is routinely accepted according to International Commission on Radiation Units (ICRU) recommendations. However, the acceptable inhomogeneity dose within the gross tumor volume (GTV) has yet to be determined (13).

The high incidence of in-field local recurrences, limitations in defining tumor areas based on MRI, lack of improvement in radiotherapy outcomes, and the results of preclinical studies suggest that sublethal dosing might promote progression. We hypothesized that parts of the GTV revealed by dtpFET-PET are treated with doses below 60 Gy, which may result in particularly high-risk areas of progression. We therefore analyzed high-dose homogeneity in the tumor region defined by dtpFET-PET and its association with location and time of progression.

## Materials and Methods

### Patients and Imaging

The dosimetric analysis was performed on 29 consecutive patients (mean age 52.4 ± 19.2) prospectively recruited to a previously published study analyzing the role of dtpFET-PET on treatment planning of newly diagnosed GBM (12). Main inclusion criteria were as follows: histological confirmation of tumor, WHO performance status 0–2, no previous radiation, aged 18 years or older, concurrent temozolomide treatment, and Karnofsky performance status >60. All patients were treated with intensity-modulated radiotherapy using the Eclipse treatment planning workstation. The total dose was 60 Gy prescribed to the ICRU Reference Point (13).

Details of dtpFET-PET and MRI imaging and target volume definitions are described in Ref. (12). The medical physicist in time of treatment planning was blinded to PET results. The plans were optimized to cover MRI-based PTV by 95–107% of prescribed dose, not the PET tumor volumes. We compared doses delivered to the site of failure with pre-irradiation images in 23 patients. Two patients did not progress, and four were lost to follow-up.

As received PTV doses range between 95 and 107% of prescribed dose, high-dose (HD) areas and suboptimal dose (SubD) areas were defined, and their volumes determined and compared with better understand heterogeneity within PTV. Assumption that doses of 57–59.9 Gy are suboptimal was taken arbitrary by dividing whole dose range within PTV.

### Dose Map Analysis at the Site of Recurrence

**Figure 1:**
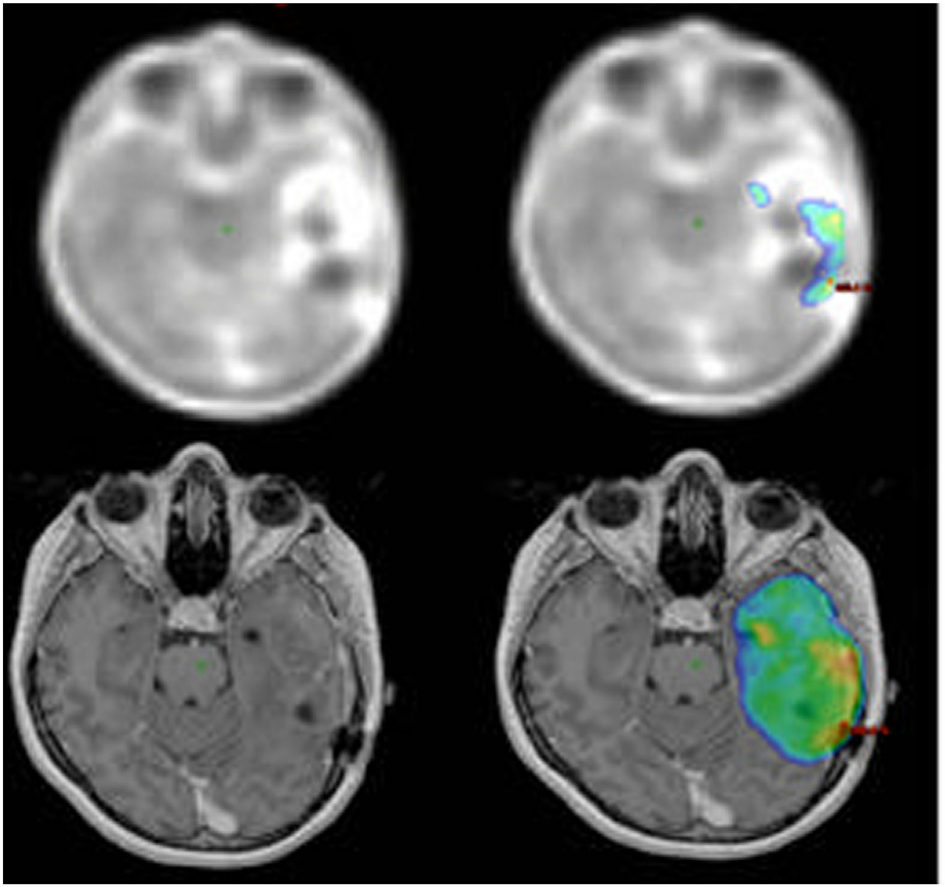
Increased 18F-FET uptake in the PET imaging (upper left) and 100% isodose map (top right). T1-gad MRI image (bottom left) and 95% isodose map (bottom right).

Difficult-to-define tumor boundaries in MRI images and well-defined boundaries in PET images are shown in the lower and upper rows of Figure 1, respectively. Balancing target volume irradiation and sparing organs at risk results in heterogeneity or cold spots within a tumor. GTV irradiated with at least 100% of the prescribed dose was defined as V100% GTV_PET_ by dtpFET-PET, V100% GTV_MRI_ by MRI, and V100% GTV_PETMRI_ for dtpFET-PET + MRI. The minimum dose in the GTV_PETMRI_ was assessed and associated with progression-free survival (PFS), and V100% results for each GTV type were correlated with PFS.

### Definitions of Progression

The time to progression was assessed as the time from the start of radiotherapy to the date when the new lesion or progressive recurrent GTV occurred on MRI. All the assessments were prospective and were blinded to original plans. Progression was determined using RANO criteria. Pseudo-progression was excluded by diffusion and perfusion MRI or FET-PET. In patients where progression was determined *via* PET, additional retrospective assessments of consecutive MRIs were undertaken to confirm progression. In 15 cases, histopathological confirmation of progression was obtained.

The following types of progression were distinguished: (i) progression in the HD area: a new lesion or increased primary tumor size within the HD area; (ii) progression in the SubD area: within the isodose 95–99.9% (57–59.9 Gy); (iii) progression in the area under prescribed dose (UD): a new focus in the dose area <95% (<57 Gy) but >50 Gy and at least stabilization in the central area; and (iv) progression in the low dose (LD) area: progression within dose <50 Gy. If progression was found in both the HD and SubD areas, the area in which the dominant tumor was located determined the type of progression.

To assess whether progression type depended on dose volume, several tests were performed: (i) the volume of each dose (for example, SubD volume) was defined in cases of progression within this dose and compared with volumes of the same dose when progression was not present; (ii) the difference between SubD volume and HD volume; and (iii) the probability of each progression type with increasing difference. Progression type according to results of V100% for GTV_PET_, GTV_PETMRI_, and GTV_MRI_ were also evaluated.

The study was performed in accordance with the principles of the Helsinki Declaration, and the Ethics Committee of Collegium Medicum of Nicolaus Copernicus University approved the protocol. All patients provided written informed consent before each FET-PET investigation.

### Statistical Analysis

Statistical analysis was performed using IBM SPSS 24.0 (IBM Inc., Chicago, IL, USA). The Shapiro–Wilks test was used to assess the normality of distributions. The relationships between variables were assessed using Spearman’s correlation coefficients. The significance of differences in the level of two quantitative variables with normal distributions was tested using the *t*-test for dependent samples. The limit of statistical significance was set at *p* < 0.05.

## Results

### Overall Coverage

**Figure 2:**
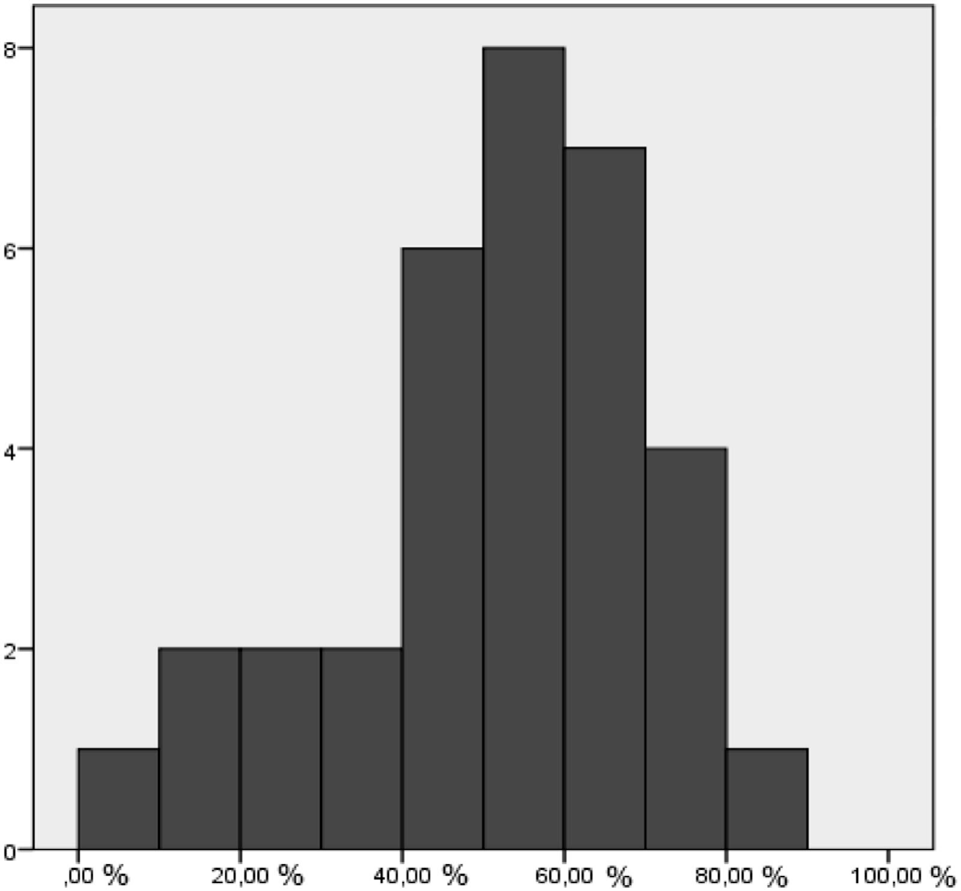
% of GTV_PETMRI_ covered by high dose in whole study group.

In no case, did the HD cover the entire GTV_PETMRI_; in most cases, the HD covered about 50% of the GTV_PETMRI_ (Figure 2). The mean suboptimal dose (SubD) volume was 143 cm^3^ and was greater than the HD volume of 108 cm^3^ (*t*-test, *p* < 0.0005).

### Evaluation of Pre-Irradiation Dose Maps Co-Registered with MRI at Time of Progression

**Figure 3:**
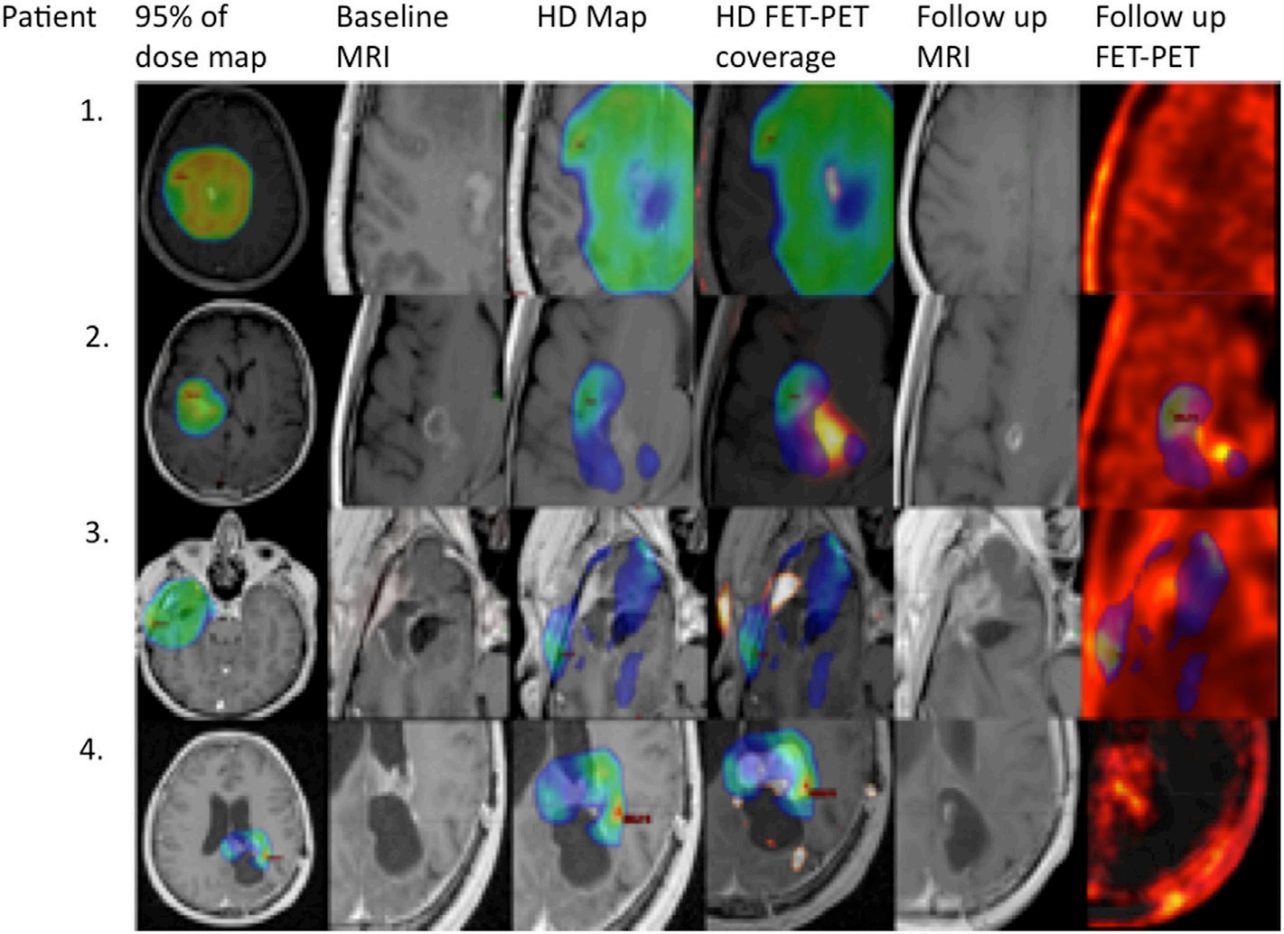
Primary fluoroethylthyrosine-PET (FET-PET) revealed an active area that was not irradiated with high dose (HD), representing a progression starting point. Areas irradiated with HD responded to treatment. From the left: primary MRI with 95% dose map, primary MRI, primary MRI with HD map, primary FET-PET/MRI with HD map, MRI at time of progression, and FET-PET at time of progression. Upper row: an example of a patient with high 100% V60 coverage of gross tumor volume (GTV)_PET_ and no progression 41 months after treatment. The second example is a patient with a V60 of GTV_PET_ of 83% and progression-free survival (PFS) of 32 months. The third example is a patient with a V60 of 45% and PFS of 7 months, and the bottom row is a patient with a V60 of 75% and PFS of 7 months.

To date, 27 new tumor foci or increased size of lesions have been identified in 23 patients (Table 1). Eight patients were lost to follow-up, and three cases were not treated with the full dose. Recurrences were significantly more frequent in the SubD area (chi-squared test, *p* = 0.004), with progression localized to the HD area in only four cases (15%). In 19 cases (56%), progression began in the SubD area, in 2 cases (7%) progression was located to the HD area, and in 6 cases (22%) progression was localized to the LD area. In 4/23 patients (17%), the new lesion was localized only to the LD area. Examples of tumor control in the HD area and progression in the SubD area are shown in Figure 3.

**Table 1:** Summary of patient data (volumes in cubic centimeters).

		*n*	Mean	Median	SD	Min	Max
Surgery	Gross total	7					
	Subtotal	18					
	Biopsy	4					

Progression	Within						
	High dose (HD)	3					
	SubD	15					
	UD	2					
	Low dose	6					

Gross tumor volume (GTV)_PETMRI_	cm^3^	29	42.1	30.4	30.9	2.8	129.2

V_60Gy_	GTV_PETMRI_	29	48.4%	50.0%	19.9	11.7%	98.5%
	GTV_MRI_	29	46.8%	47.6%	21.4	3.5%	98.5%
	GTV_PET_	29	47.4%	50.0%	22.1	2.5%	100.0%

HD volume	cm^3^	29	108.3	108.3	48.1	14.8	207.8

SubD volume	cm^3^	29	143.4	140.9	62.5	16.6	266.7

*D*_min_	Gy	29	54.9	56.5	5.0	36.3	59.3

Progression-free survival	Months	25	10.1	7.0	10.1	2	41

### Correlation between V100% Results and Time to Progression

**Figure 4:**
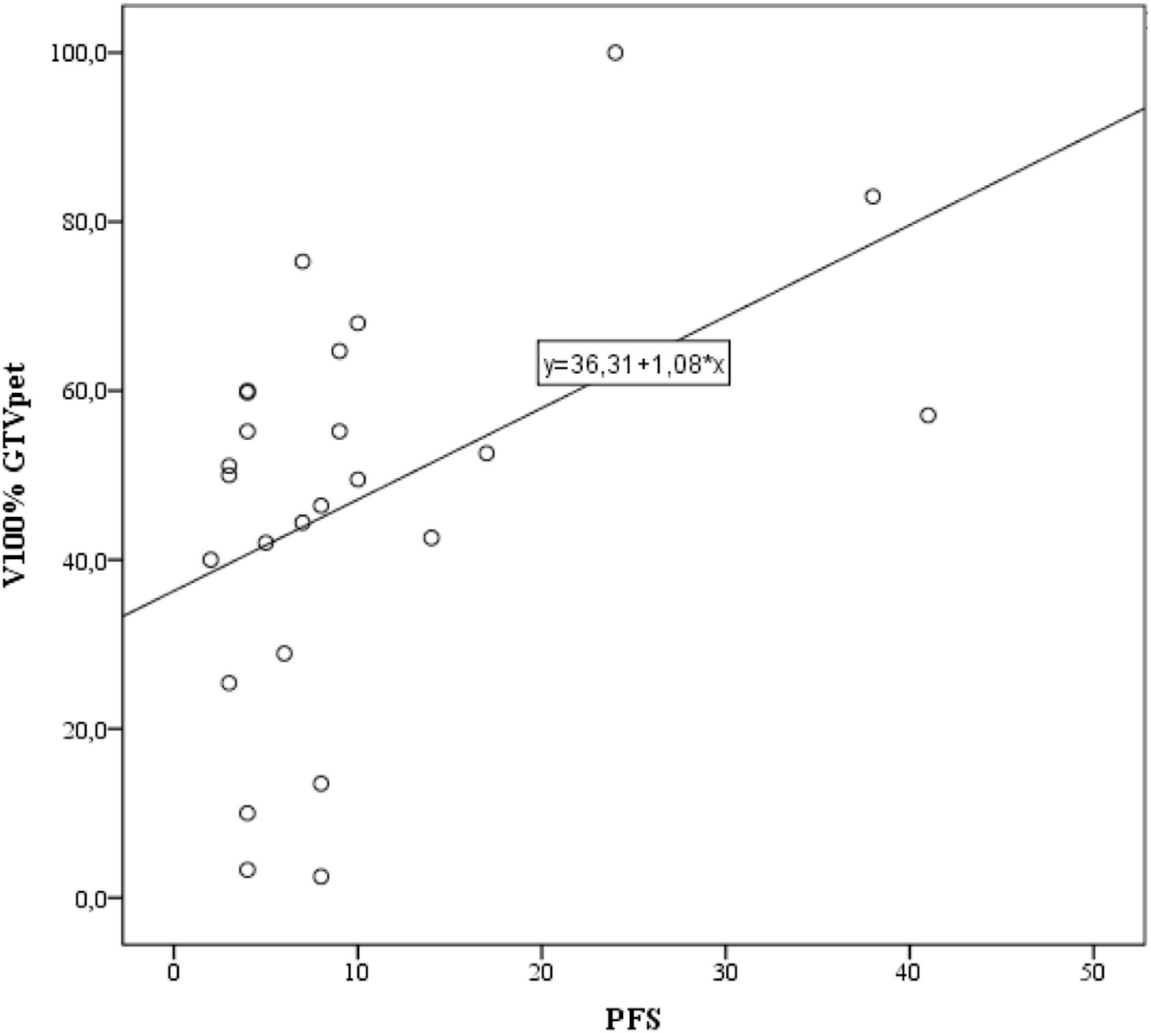
Correlation between V100% gross tumor volume (GTV)_PET_ and progression-free survival (PFS). Simple linear regression performed using the least squares method with the regression function shown.

There was a positive correlation between V100% for GTV_PET_ and PFS (Spearman’s rho = 0.417; *p* = 0.038; Figure 4). There was no relationship between V100% GTV_MRI_, V100% GTV_PETMRI_, *D*_min_ within GTV_PETMRI_, and PFS.

### Progression Type vs. Dose Volumes

Progressions occurred more frequently inside the SubD area at lower volumes of this area (*p* = 0.024; Table 2). There was no relationship between HD volume and progression in or outside this area. Cases with progression inside the SubD area were significantly more common when the differences between SubD and HD volumes were smaller (Mann–Whitney *U*-test, *p* = 0.01). The HD and SubD volumes were similar regardless of progression type. Evaluation of progression type according GTV_PET_, GTV_PETMRI_, and GTV_MRI_ V100% revealed that there was no relationship between coverage of GTVs by HD and progression type when evaluated by receiver-operating characteristics curves. In the case of distant progression, the median coverage was highest and amounted to 60% of the GTV_PETMRI_ area, and in the case of multifocal progression the smallest (18.5%).

**Table 2:** Comparison of isodose SubD volume and difference between SubD and high-dose (HD) volumes in cases of progression.

	Mean	Median	SubD	Minimum	Maximum	*N*
**SubD volume**						
No progression within SubD	186.2	178.7	44.6	137.8	263.5	8
Progression within SubD	123.6	132.6	66.7	16.6	266.7	15
Overall	145.4	140.9	66.3	16.6	266.7	23

**SubD volume–HD volume**						
No progression within SubD	54.2	55.8	10.6	30.8	63.9	8
Progression within SubD	30.5	36.5	48.4	−110.3	133.3	15

## Discussion

Glioblastoma multiforme is characterized by extensive infiltration that is often invisible on MRI. Geographical error is frequently cited as one of the main reasons for failure of dose escalation trials. dtpFET-PET can be used to more accurately detect and map infiltration (12). Here, we show with molecular imaging that increased, higher dose coverage of dtpFET-PET tumor regions is positively correlated with time to progression. In addition, suboptimally treated areas were the most common site of progression irrespective of the HD and SubD volume difference.

We have recently reported significant inhomogeneity in planning target volumes even with IMRT planning, resulting in approximately 50% coverage of GTV_PETMRI_ areas irradiated with at minimum 60 Gy. It must be emphasized that only adequate coverage of the PTV and not GTV_PETMRI_ (according to ICRU reporting) was analyzed when selecting treatment plans. Such high heterogeneity can result from highly complex geometric configurations and frequent localization in the proximity of the organ at risk. The tumor shape is more complicated when defined by PET. It has previously been shown that IMRT treatment planning for GBM defined by PET achieves better homogeneity and conformity compared with 3D-CRT, especially in the case of subvolumes (14).

Local recurrences following conventional radiotherapy are still the most common cause of therapeutic failure. Local control has not been successfully achieved by intensive therapy protocols such as accelerated treatment (15), escalated total dose (6), escalated dose per fraction (16, 17), or by adding a boost (18, 19). The distribution of progressions in our study indicated that the most frequent location of the surviving cell fraction was in the 95–99% dose area, especially if it coincided with the original GTV_PET_ or GTV_MRI_ image.

Mathematical models have shown that the administration of an additional dose to areas at higher risk of recurrence should be beneficial to the outcome of treatment of malignant gliomas (20). However, it is difficult to determine how high the dose should be. It has been suggested that to overcome the radioresistance of GBM, the dose should be several times higher than currently administered (21). In preclinical studies, a lack of significant migration and invasion of human U87MG cells after 16 Gy delivered as a single fraction could indicate the possibility of local tumor growth control with further increases in dose (8).

Clinical trials have tested doses of less than 100 Gy. Lee et al. (5) used a dose escalation protocol of 70–80 Gy on GTV defined by MRI; 35/36 patients did not achieve local control in the HD irradiated area. In another escalation dose study up to 90 Gy, very good local control was observed with progression at lower-dose sites; only 1/23 progressions in 90 Gy patients occurred in the HD irradiated area, with the majority of recurrences occurring in the 70–80 Gy area and extended overall survival compared with historical controls (22). However, another study of 23 patients with GBM progression with escalation up to 90 Gy failed to observe a similar effect (6). 78, 13, 9, and 0% of failures were localized in the HD field, field boundary, and distal brain structures from the irradiated field, respectively. Despite the high dose in the MRI area, all progressions were in contact with the irradiated field.

This difference between studies in terms of local control after HD irradiation may be due to errors in tumor definition using MRI in individual patients. GTV_PET_ and GTV_MRI_ analyses (12, 23, 24) have shown that distance between contours can reach up to 35 mm. In addition, sites seen in PET before radiotherapy are the most likely site of failure (12, 25). Weber et al. (26) analyzed progression in 10 patients irradiated to 60 Gy with respect to MRI and PET tumor volumes and found that 90% of progressions were in the dose area >95%. A 60 Gy dose regimen to GTV_PETMRI_ does not appear to provide optimal local control.

We noted a correlation between GTV_PET_ V100% and PFS. Our results may suggest that the better the 60 Gy coverage of GTV_PET_, the better the local control. GTV_PET_ best illustrates active tumor while GTV_PETMRI_ and GTV_MRI_ also contain inactive parts such as a cerebrospinal fluid in the tumor bed. Our findings are in line with previous suggestions (27) that localized treatment failure may be determined by the minimum dose within the tumor. It has also been shown that inaccurate coverage of areas changed in PET images results in more frequent progressions in these areas (25). The results do not support the thesis that any dose may cause recurrences (7, 8), but to some extent confirm that the smaller the dose deposited in the tumor, the faster the progression. This is consistent with traditional radiobiological dogma that a single malignant tumor cell that has not been treated with a lethal dose can cause therapeutic failure.

The high incidence of progression in areas associated with GTV_PET_ reported previously (12) suggests that dtpFET-PET treatment planning may improve results. One possible solution may be dose escalation for areas with increased FET uptake. Few studies have tested dtpFET-PET dose escalation. Piroth et al. (14) adopted the concept of PET-based dose escalation using static FET-PET examination. Both strategies resulted in different GTV shapes and volumes. In the Piroth study, if the minimum and maximum doses in the PTV1 (GTV_PET_ + margin) areas were within the range 60–72 Gy, the mean minimum was 51.4 Gy (86% prescribed dose) in the PTV2 area (GTV_RM_ + 20 mm margin). All recurrences were observed in the area of 95% of the 60 Gy dose, corresponding to the SubD area in our study (14). It has been suggested that the minimum dose may determine local control (28), and our results seem to confirm this. In addition, in this context, the method of GTV_PET_ definition may be significant (29). Piroth et al. (14) used a definition 40%× SUV_MAX_, which leads to different GTV volumes to our method and proposed by Pauleit et al. (10). In our experience, 1.6× SUV_MEAN_ usually results in a larger area than 40%× SUV_MAX_. Determining the GTV_PET_ area best suited to actual glioblastoma infiltration may be crucial in future PET-based irradiation studies. Overcoming GBM resistance appears to be possible with dose escalation only after optimal tumor border identification.

We still noted marginal and distant failures. It remains unclear how many of the new lesions occurring out of the irradiated field would have been prevented by improving local control. Due to increasing treatment margins, there is a loss of high and homogenous dosing within the treatment volume, especially in the proximity of organs at risk. In addition to the target complexity discussed earlier, this trade-off becomes even more important. In the chemoradiotherapy era, reducing margins (RTOG edema + 2 cm vs. EORTC contrast enhancement + 2 cm) may not affect treatment outcomes (30). However, there is lack of evidence on how far margins can be reduced by without losing efficacy. FET-PET-based planning shows promise and provides the first evidence that by incorporating FET uptake we can irradiate most possible site of failure (12). In one study, after whole-brain irradiation, there were no distant progressions in cases where there was no progression in the primary focus (31).

In general, there is considerable discrepancy in frequency of out-of-field recurrences between studies. Our study results are poor; in 17% of patients or in 22% of new lesions, distant progression was the only progressive site. However, such a high rate of out-of field progression is consistent with previous recurrence studies after involved field irradiation of 60 Gy dosing with temozolomide (32) or higher total doses (28). In another retrospective analysis, 40–60 Gy WBRT with a boost to the restricted field had 78% local progressions and 22% out-of-field progressions (33). By contrast, no distant failures were observed using CT-based radiotherapy alone (34), CT/MRI based (5), or total brain irradiation (4, 31). One possible explanation for this is that the most distant progressions due to poor local control are unlikely to manifest clinically. It has recently been shown that the rate of distant progression may increase with prolongation of survival (35).

Nevertheless, there was no statistically significant difference between the 60 Gy dose coverage of GTV_PET_ and the type of progression, with the highest tumor coverage noted in patients with distant failures and the smallest in uncontrolled multifocal recurrences. However, low numbers limit the analysis. Longer overall median survival may also explain more frequent out-of-field progressions in the MGMT group (36). We did not perform MGMT analysis because it is not standard of care in Poland. Therefore, proper selection of patients with increased risk of distant recurrence (such as primary dissemination to cerebrospinal fluid or intrinsic tumor biology) may be crucial in dose escalation trials. Otherwise, improved disease control in the irradiated area may have less of an impact on the final outcome in approximately 15–30% of patients with primary GBM.

Although the small group is a limitation of our study, the strict selection criteria for the study population are likely to have improved the data quality. This result needs confirming in a larger, prospective cohort. Moreover, data from patients who received a rather large coverage of their tumors and still progressed early serves to remind us that PET coverage are not the only predictive factor of radiotherapy outcome.

In spite of this being a relatively small and retrospective analysis, this is the first to use dtpFET-PET and a homogenous group of glioblastoma patients irradiated with 60 Gy and followed-up with FET-PET. Even if the lethal dose is applied to part of the tumor mass, glioma cells located outside this area may receive sublethal doses, leading to further growth and recurrence. Our results suggest that suboptimal dosing within areas of increased uptake in dtpFET-PET is at high risk of GBM recurrence. Moreover, underdosing represented by V100% of GTV_PET_ may correlate with PFS. However, this observation requires confirmation in a larger dataset and in parallel with well-established prognostic glioma markers.

## Author Contributions

MH conceived and designed the experiments; MH and BM performed the experiments; MH and IW analyzed the data; MH and KR contributed reagents/materials/analysis tools; MH and RM wrote the paper.

## Conflict of Interest Statement

The authors declare that the research was conducted in the absence of any commercial or financial relationships that could be construed as a potential conflict of interest.
